# A method for cryo-EM analysis of eukaryotic nucleosomes reconstituted in bacterial cells

**DOI:** 10.1016/j.isci.2025.114453

**Published:** 2025-12-16

**Authors:** Cheng-Han Ho, Yuki Kobayashi, Mitsuo Ogasawara, Yoshimasa Takizawa, Hitoshi Kurumizaka

**Affiliations:** 1 Laboratory of Chromatin Structure and Function, Institute for Quantitative Biosciences, The University of Tokyo, 1-1-1 Yayoi, Bunkyo-ku, Tokyo 113-0032, Japan; 2 Department of Computational Biology and Medical Sciences, Graduate School of Frontier Sciences, The University of Tokyo, 1-1-1 Yayoi, Bunkyo-ku, Tokyo 113-0032, Japan; 3 Department of Biological Sciences, Graduate School of Science, The University of Tokyo, 1-1-1 Yayoi, Bunkyo-ku, Tokyo 113-0032, Japan; 4 RIKEN Center for Integrative Medical Sciences, 1-7-22 Suehiro-cho, Tsurumi-ku, Yokohama 230-0045, Japan

**Keywords:** biochemistry, molecular biology, structural biology

## Abstract

Conventional methods for preparing nucleosomes are time-consuming and technically demanding. In the present study, we extended the approach of generating nucleosomes in *Escherichia coli* by the co-expression of all four histones, allowing nucleosomes to be assembled in cells. The bacterially reconstituted nucleosomes can be readily prepared from the *E. coli* cells and directly subjected to cryo-EM single particle analysis. Using this method, we obtained a 2.56 Å nucleosome structure that is highly similar to a previously reported nucleosome crystal structure, validating the use of nucleosomes formed in *E. coli* for cryo-EM analysis. Unexpectedly, we also discovered a non-canonical nucleosome structure, in which two hexasomes are closely packed. This system provides a robust and efficient platform for structural studies of nucleosomes and nucleosome variants, and may facilitate the discovery of chromatin architectures.

## Highlights

• Nucleosomes assembled within *E. coli* cells can be used for cryo-EM analysis
• Nucleosomes formed in *E. coli* cells closely resemble those reconstituted *in vitro*
• The close-packed di-hexasome (CPDH) structure is discovered

## Graphical abstract

**Figure:**
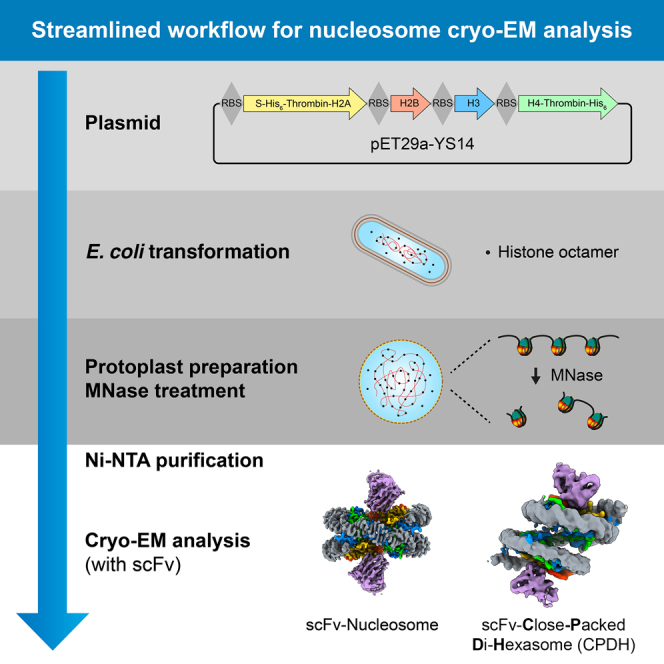

## Introduction

In eukaryotes, the nucleosome serves as the fundamental unit for packing genomic DNA within the cell nucleus. The nucleosome is composed of approximately 145 bp of DNA wrapped around a histone octamer, which consists of two molecules each of the four core histones (H2A, H2B, H3, and H4). The nucleosome is involved in various nuclear activities, such as transcription,^1^^,^^2^^,^^3^ DNA replication, and DNA damage repair,^4^ making it a prominent focus of structural analysis in chromatin biology. For structural analysis, relatively homogeneous nucleosomes are required. Although nucleosomes are abundant in eukaryotic cells, those nucleosomes typically contain histone variants, as well as histones that are post-transcriptionally modified. This increases the heterogeneity of the nucleosomes, presenting challenges to structural analyses as well as interpretations of the results.

To overcome this issue, individual histones can be expressed as recombinant proteins in bacterial cells, which lack endogenous histones.^5^ Subsequently, the individual histones are purified under denaturing conditions, before being combined to reconstitute the histone octamer *in vitro*. The histone octamer is then mixed with a DNA fragment and reconstituted, by methods such as salt dialysis, to form the nucleosome. This approach guarantees highly purified nucleosomes: It has been proven effective in many biochemical and structural studies to date, including the recently reported 1.89 Å nucleosome cryo-EM structure.^6^ Nevertheless, this “conventional method” of nucleosome preparation is time-consuming and technically difficult, prompting us to seek new approaches to streamline the nucleosome preparation process for cryo-EM analysis.

Recent studies have provided hints for a new workflow. These reports described the formation of nucleosome-like particles in the *Escherichia coli* genome, following the co-expression of all four core histones.^7^^,^^8^ Unfortunately, no molecular structures of these putative nucleosomes formed in the *E. coli* cells were reported, and thus further clarification is required. Inspired by our previous ChIP-cryoEM analysis,^9^ we sought to explore the possibility of performing cryo-EM analysis on nucleosomes generated in *E. coli*. This could provide structural evidence for previous work, as well as a new workflow for nucleosome cryo-EM analysis.

In the present study, we transformed a polycistronic plasmid that could co-express all four *Xenopus* *laevis* core histones^10^ into the *E. coli* strain BL21(DE3). We confirmed the formation of nucleosomes by MNase digestion and isolated them by Ni-NTA affinity purification. Finally, we performed a cryo-EM analysis and obtained a high-resolution 2.56 Å structure, which clearly demonstrates the formation of nucleosomes in *E. coli* cells. Importantly, the nucleosome structure obtained in this study is almost identical to a previously reported nucleosome crystal structure,^11^ validating this new nucleosome preparation workflow. Unexpectedly, we also obtained the structure of a subnucleosome, the close-packed di-hexasome (CPDH), in which two hexasomes are closely associated.

## Results

### Purifying nucleosomes reconstituted in *E. coli* cells

**Figure 1:**
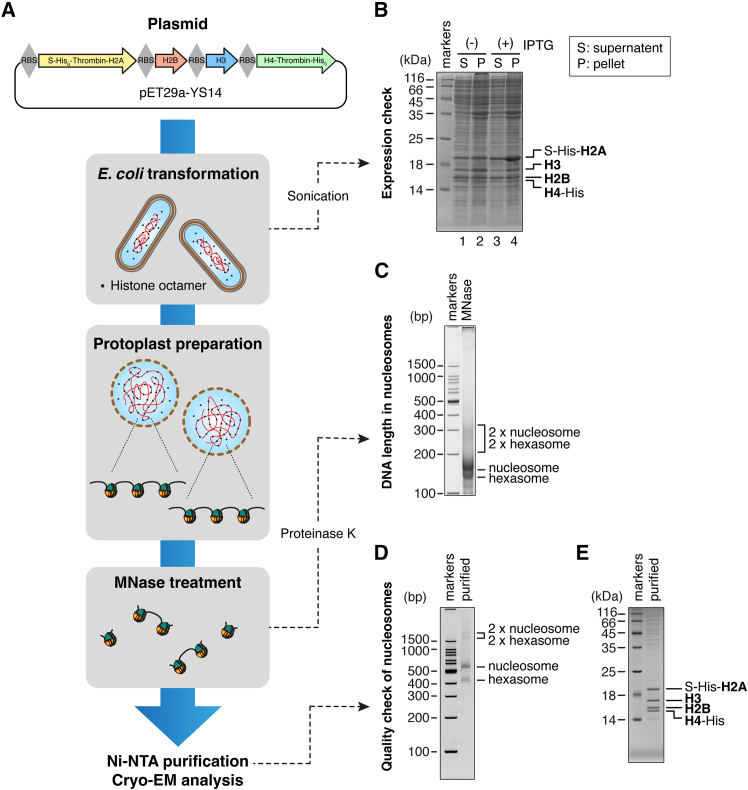
Workflow for purifying nucleosomes formed in *E. coli* cells (A) Overview of the workflow. (B) Expression check of the four histones after sonication. The samples were analyzed by SDS-PAGE. (C) DNA fragmentation profiles of the *E. coli* cells. The sample was analyzed by native PAGE. (D and E) Quality checks of the purified nucleosomes by native PAGE (D) and SDS-PAGE (E).

To prepare the “potential nucleosomes” reconstituted in the *E. coli* cells, we first optimized the conditions for co-expressing all four core histones. We utilized the pET29a-YS14 plasmid, which encodes a polycistronic construct for co-expressing S-tag-His_6_-H2A, H2B, H3, and H4-His_6_ (Figure 1A, top). This plasmid is designed to facilitate the non-denaturing purification of the histone octamer.^10^

The plasmid was transformed into BL21(DE3) cells. After harvesting and sonicating the cells, we analyzed histone expression by SDS-PAGE (Figure 1B). The histone bands were identified on the SDS gel by comparing the bands with those in previous reports, including the original paper describing the pET29a-YS14 plasmid,^10^ as well as more recent reports on nucleosomes forming in *E. coli* cells.^7^^,^^8^ The bands identified in this study correspond well to those in the previous studies, and to the theoretical molecular weights of histones (S-His_6_-H2A: 18.0 kDa, H2B: 13.6 kDa, H3: 15.4 kDa, and H4-His_6_: 13.3 kDa). All four histones were detected under both isopropyl β-D-1-thiogalactopyranoside (IPTG)-induced and uninduced conditions, indicating that the co-expression of H2A, H2B, H3, and H4 is achievable without IPTG. Notably, the histones were present in the soluble fraction under both conditions (Figure 1B, lanes 1 and 3), validating that recovery without denaturation is possible. Interestingly, the uninduced conditions yielded more uniform expression levels for all four histones (Figure 1B, lanes 1 and 2). As the overexpression of H2A and H2B relative to H3 and H4 could potentially hinder proper histone octamer formation, we chose to proceed with the conditions without IPTG induction for subsequent experiments.

In the previous study, nucleosomes were purified from *E. coli* cells by sonication combined with Ni-NTA affinity chromatography.^7^ However, as it is challenging to maintain the reproducibility of sonication, we aimed to establish a workflow based on MNase digestion. A small-scale MNase digestion check could be performed to determine the optimal MNase concentration, before digesting the rest of the sample.

To this end, we combined the previously reported *in situ* MNase digestion protocol for *E. coli* (the ecMNase assay)^7^ with Ni-NTA affinity purification. As shown in Figure 1A, protoplasts were first generated to permit MNase entry and digestion of the intracellular DNA. Following MNase treatment and deproteinization with Proteinase K, we observed DNA fragments between the 100 and 200 bp markers, corresponding to nucleosomes, along with a slightly lower band likely representing hexasomes (Figure 1C). A faint smear between the 200 and 300 bp markers was also detected. These results suggest that not only histone octamers but also fully assembled nucleosomes were successfully formed within the *E. coli* cells.

Finally, we proceeded to purify these nucleosomes. Since H2A and H4 were tagged with His_6_, we employed Ni-NTA affinity chromatography for purification. After buffer exchange, we successfully obtained nucleosomes and hexasomes, primarily migrating near the 400–600 bp markers (Figure 1D). Interestingly, weaker bands were also observed slightly above the 1500 bp marker, which may represent di-nucleosomes or other non-canonical nucleosomal structures. SDS-PAGE analysis revealed a quite clean preparation, with distinct bands corresponding to all four histones (Figure 1E). These results encouraged us to proceed with the cryo-EM single particle analysis.

### PL2-6 binding indicates successful nucleosome formation

In cryo-EM single particle analysis, protein complexes are frequently damaged by the air-water interface (AWI). A previously reported single-chain variable fragment (scFv), PL2-6, can effectively shield nucleosomes from AWI-induced damage by binding with high affinity to the nucleosome’s acidic patch.^6^^,^^12^^,^^13^^,^^14^ This patch is formed on the H2A-H2B surface of the fully assembled nucleosome, and thus PL2-6 binding can also serve as an indicator of proper nucleosome formation.

**Figure 2:**
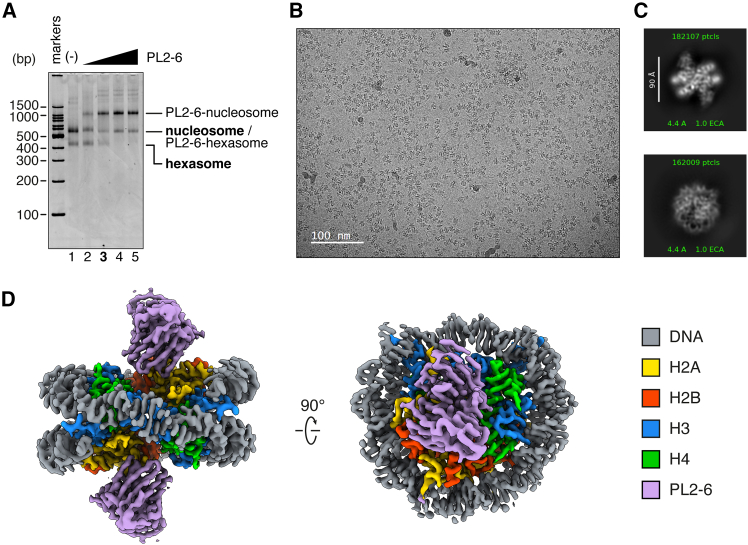
Cryo-EM analysis of PL2-6-bound *E. coli*-derived nucleosomes (A) Electrophoretic mobility shift assay (EMSA) of PL2-6 and the nucleosomes purified from *E. coli*. (B) Representative micrograph of the PL2-6-bound *E. coli*-derived nucleosome. Scale bar indicates 100 nm. (C) Representative 2D class averages from CryoSPARC.^15^ (D) Cryo-EM structure of the PL2-6-bound *E. coli*-derived nucleosome with a resolution of 2.56 Å. The map was sharpened by DeepEMhancer^16^ and visualized in ChimeraX.^17^

To determine the optimal amount of PL2-6 required and further validate the nucleosome formation in the *E. coli*-derived sample, we performed a binding assay using PL2-6 (Figure 2A). Upon increasing the concentration of PL2-6, the major nucleosome band (migrating between 500 and 600 bp markers) exhibited an upward mobility shift. The lower main band (between 400 and 500 bp markers), possibly corresponding to the hexasome band, diminished with increased PL2-6. It is possible that PL2-6 binding to hexasomes resulted in co-migration with the nucleosome band; however, this could not be conclusively determined. Notably, the weak bands migrating above the 1500 bp marker also shifted upon PL2-6 addition, implying that these populations contain exposed acidic patches and are capable of interacting with the scFv. In conclusion, the binding assay further supported successful nucleosome formation, and we selected the optimal PL2-6 concentration (Figure 2A, lane 3) for the subsequent single particle cryo-EM analysis.

### High-resolution cryo-EM structure of the nucleosome formed in *E. coli* cells

After preparing cryo-EM grids with the PL2-6-bound nucleosomes from the *E. coli* cells, we performed an initial screening (Figure 2B). The micrographs revealed an abundance of nucleosome-like particles, prompting us to proceed with data collection and single particle analysis. During the 2D classification, extremely clear classes corresponding to PL2-6-bound nucleosomes were already visible (Figure 2C). Ultimately, we obtained a high-resolution structure of a PL2-6-bound nucleosome at 2.56 Å resolution (Figures 2D, S1, and S2; Table S1).

**Figure 3:**
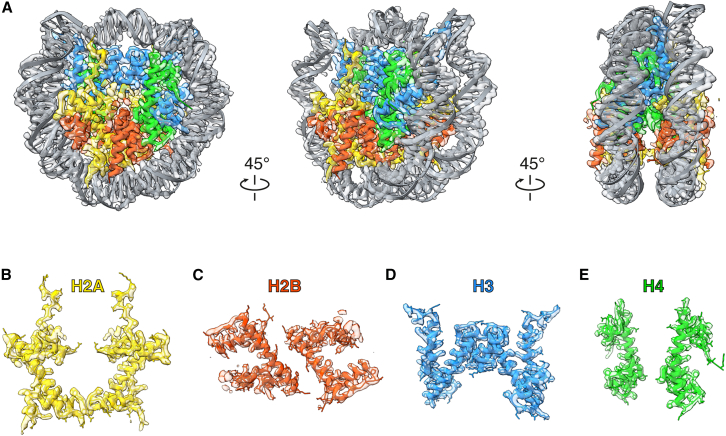
Comparison of the *E. coli*-derived *Xenopus* nucleosome with the previously reported *Xenopus* nucleosome crystal structure (A) The crystal structure of the *Xenopus* nucleosome (PDB: 3LZ0) is fitted into the cryo-EM map in Figure 2D. (B–E) Comparison of the cryo-EM map to the crystal structure (PDB: 3LZ0), showing H2A (B), H2B (C), H3 (D), and H4 (E). All panels were visualized in ChimeraX.^17^

At first glance, the overall architecture of the *E. coli*-derived nucleosome closely resembled that of a “normal” nucleosome (Figure 2D). We next fitted the crystal structure of a reconstituted *Xenopus laevis* nucleosome containing the Widom 601 DNA sequence^11^ into our cryo-EM map (Figure 3A). Despite the fact that the *E. coli*-derived nucleosomes in this study were assembled on the *E. coli* genomic DNA, rather than a defined sequence, the DNA path was quite similar to that of the 601-based structure. The high-resolution map enabled the clear visualization of the side chains of all four histones (Figures 3B–3E). Not only did the histone backbones align excellently with those in the crystal structure, but the side chains also showed good correspondence. The cryo-EM map of the *E. coli*-derived *Xenopus* nucleosome obtained in this study also corresponds well to that of an *in vitro* reconstituted *Xenopus* nucleosome^18^ (Figure S3).

Based on these results, we conclude that the nucleosomes assembled in *E. coli* are structurally indistinguishable from those reconstituted *in vitro*, demonstrating that this *E. coli*-based system is well-suited for high-resolution cryo-EM analyses.

### Discovery of a subnucleosome structure

**Figure 4:**
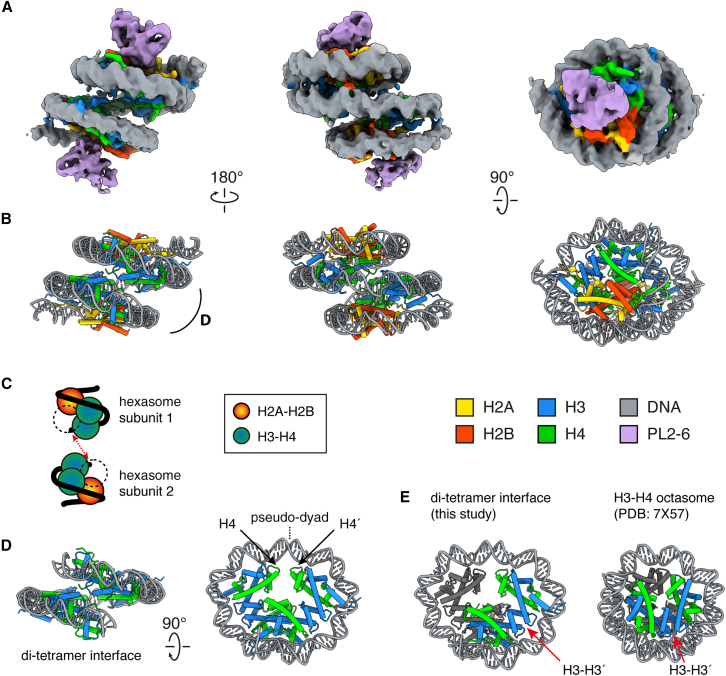
Structure of the close-packed di-hexasome (CPDH) (A) Cryo-EM map of the CPDH. (B) Atomic model of the CPDH. (C) Schematic representation of the CPDH, with the same orientation as in the left image of Figure 4B. (D) The center di-(H3-H4)_2_ tetramer part of the CPDH is extracted from the full model. The pseudo-dyad position is also illustrated. (E) Comparison of the center part of the CPDH in Figure 4D with the H3-H4 octasome. Both structures are aligned by the left-bottom (H3-H4)_2_ tetramer, which is colored gray. The H3-H3′ four-helix bundle is also depicted. All panels were visualized in ChimeraX.^17^

During the single particle analysis, we identified several classes resembling hexasomes. To confirm their identity as hexasomes and verify the absence of other subunits, we performed further classification. Surprisingly, we identified a special class that resembled an overlapping di-nucleosome (OLDN)^19^ (Figures 4A, S1, and S2; Table S1). However, a closer inspection revealed the true identity of this unique structure: two extremely closely packed hexasomes (Figures 4A and 4B).

In this structure, two PL2-6 molecules are associated with the top and bottom sides of the OLDN-like structure, indicating that the H2A-H2B dimers are present on both sides (Figures 4A–4C). We refer to this previously unreported, non-canonical nucleosomal particle as the close-packed di-hexasome (CPDH), and it contains 207 bp of DNA, which is shorter than the 250 bp of DNA seen in the OLDN. In contrast to the OLDN, the CPDH lacks both H2A-H2B dimers at the interface between the two hexasome units, making the nucleoprotein complex symmetrical about the pseudo-dyad, if not considering the DNA sequence (Figure 4D).

The center of the CPDH structure features two closely positioned (H3-H4)_2_ tetramers (Figure 4D). To further investigate the central region of the CPDH, we compared it with the related H3-H4 octasome, a nucleosome-like particle composed of two (H3-H4)_2_ tetramers.^20^ The structural comparison revealed that the central region of the CPDH is more open than that of the H3-H4 octasome, with greater separation between the H4-H4 four-helix bundles and the DNA adopting an oval-shaped trajectory (Figure 4E, left image). Importantly, the relative (H3-H4)_2_ tetramer positions are also quite different: when aligning both structures to the “left” tetramer unit, as in Figure 4E, the “right” tetramer unit in the CPDH adopts a more unwound conformation as compared to that in the H3-H4 octasome. Together, these findings suggest that the CPDH represents a structurally distinct non-canonical nucleosomal particle that may offer new insights into intermediate states of nucleosome assembly or remodeling.

## Discussion

The possibility of nucleosome formation in *E. coli* cells co-expressing all four core histones was demonstrated previously.^7^^,^^8^ However, the studies lacked structural evidence of such nucleosomes. Here, we extended the previous work and performed a cryo-EM analysis of the nucleosomes formed within *E. coli* cells. We obtained a high-resolution 2.56 Å nucleosome structure, which is almost indistinguishable from the previously reported crystal structure.^11^ This provides structural evidence of nucleosome formation in *E. coli*. The result also demonstrates that the nucleosomes formed in *E. coli* cells are suitable for high-resolution structural studies, providing a new workflow for nucleosome cryo-EM analysis.

Unlike conventional nucleosome reconstitution methods, in which the four histones are expressed and purified individually, and then combined with a defined DNA fragment to form nucleosomes, the workflow reported in this study is time-saving. The entire process from histone co-expression to cryo-EM grid preparation can be completed in less than 1 week. Additionally, the nucleosomes assembled on random *E. coli* genomic DNA may reflect a more natural substrate than those based on artificial sequences such as Widom 601.^21^ This approach may also inherently favor the formation of nucleosomes on the highest-affinity DNA sequences present within the bacterial genome. In this sense, the method could serve as a natural selection system for identifying DNA sequences with strong histone-binding potential. Future analyses of nucleosomal DNA recovered from *E. coli* could therefore provide valuable insights for designing or predicting DNA sequences with enhanced nucleosome-forming propensity. Another advantage of this method is that it does not require histone denaturation. Conventional protocols may not be compatible with histone variants that are unstable under denaturing conditions. Our workflow therefore offers an alternative for structural analyses of nucleosomes containing fragile or poorly folding histone variants.

Nucleosome binding factors, such as linker histone H1 or histone writers, may also be co-expressed with the four histones to form nucleosomal complexes. This workflow could save time and serve as a screening platform for determining the optimal nucleosome substrate for complex reconstitution (e.g., determining whether to use nucleosomes with or without linker DNA, selecting between mono- or di-nucleosomes, and optimizing linker DNA length for stable complex formation). This method may also be particularly advantageous for assembling nucleosomes with transient or weakly interacting factors, which are often challenging to purify using conventional methods. Crosslinking *E. coli* cells prior to MNase digestion may further stabilize such interactions.

In this study, we did not perform cryo-EM analysis on nucleosomes alone, as we employed PL2-6 to protect the nucleosomes from the AWI during cryo-EM. Based on our previous experiences, free nucleosomes without crosslinking are highly sensitive to the AWI, whereas PL2-6 binding markedly improves nucleosome integrity and enables the acquisition of optimal micrographs at lower sample concentrations. We note that the presence of PL2-6 may influence particle behavior to some extent. In particular, a degree of nucleosome orientation bias was observed in the vitrified sample, as relatively few nucleosome disc views were present in Figure 2B. Fortunately, this bias was mitigated at an early stage of the single-particle analysis workflow, allowing us to obtain a high-quality nucleosome final map. The underlying cause of this orientation bias remains uncertain: it may reflect the effect of PL2-6 binding, the intrinsic properties of *E. coli*-derived nucleosomes, the presence of minor contaminating proteins, or other factors. Since PL2-6 has been used in several previous studies^6^^,^^12^^,^^13^^,^^22^^,^^23^ and shown not to alter the overall nucleosome structure,^12^ its effect on the nucleosome structure in this study should be minimal. For future applications, alternative stabilization methods such as Gradient Fixation (GraFix)^24^ or mild crosslinking prior to MNase digestion could also be used, particularly when studying factors that bind to the acidic patch. In some cases, the binding partner itself may serve the same protective function as PL2-6.

Intriguingly, the formation of nucleosomes on the *E. coli* genome does not seem to strongly affect *E. coli* survival under normal conditions, in agreement with the previous report.^7^ This suggests that nucleosome assembly itself does not impose a strong enough barrier to completely stall *E. coli* RNA polymerase, halt transcription, and kill the *E. coli* cells. This raises interesting questions about the importance of additional regulatory factors in eukaryotic cell systems that contribute to gene repression. A reduced complexity system such as *E. coli* may serve as a valuable platform for dissecting the minimal components required for transcriptional regulation when co-expressing all four core histones and other regulatory eukaryotic proteins.

We also report the structure of a previously undocumented, non-canonical nucleosome, the CPDH. This particle consists of two hexasomes closely associated through their exposed (H3-H4)_2_ tetramers, forming a central interface devoid of H2A-H2B dimers. While the CPDH may represent an artifact of overexpression or the *E. coli* environment, it is also possible that it reflects a *bona fide* intermediate in nucleosome assembly or remodeling. Future analyses of MNase-seq data in eukaryotic cells may help to determine whether similar structures have been previously overlooked. Alternatively, the CPDH may be actively resolved by chromatin remodelers or chaperones in eukaryotic contexts. In cells, subnucleosomes such as the CPDH may function in gene regulation, but the exact function remains to be determined.

In summary, we present a streamlined workflow for nucleosome preparation in *E. coli* for cryo-EM analysis and report a subnucleosomal structure with unknown biological relevance. This workflow opens new possibilities for chromatin structural biology and may enable studies previously inaccessible by conventional reconstitution approaches. Further investigations into the CPDH and related intermediates may yield new insights into the dynamics of nucleosome assembly and remodeling.

### Limitations of the study

Despite the aforementioned advantages of this new method, certain limitations remain. The inability to control the DNA sequence restricts the use of this method from studying sequence-specific DNA-binding factors. Likewise, it is not possible to add site-specific histone modifications to the nucleosomes, making it difficult to apply to analyses of readers of the modifications. Further improvements of this method may be developed to enable the preparation of nucleosomes with DNA sequence specificity or histone modifications. A potential strategy to address this limitation would be to co-transform a plasmid-based DNA construct—such as a plasmid containing tandem 601 sequences flanked by restriction enzyme sites—with the plasmid encoding the four core histones. This additional plasmid could also include tandem *lacO* sequences, allowing the selective isolation of the plasmid through tagged LacI affinity purification and IPTG-mediated elution.^25^ After isolation, nucleosomes could be released from the plasmid by MNase digestion or restriction enzyme cleavage at the defined sites. However, compared to the long genomic DNA of *E. coli*, the plasmid DNA would provide a much shorter template, which may limit the overall yield of recovered nucleosomes.

Although OLDN structures were not observed in this study, we cannot rule out the possibility that they may also be formed. When we classified the hexasome-like particles, we observed many classes with a blob-like density near the hexasome. The addition of PL2-6 could have interfered with the stacking of the two subunits of the OLDN, leading to a flexible nucleosome subunit that could not be resolved. Future studies, using methods such as GraFix, will be necessary to investigate whether OLDNs can also be formed in *E. coli* cells.

## Resource availability

### Lead contact

Further information and requests for resources and reagents should be directed to and will be fulfilled by the lead contact, Hitoshi Kurumizaka (kurumizaka@iqb.u-tokyo.ac.jp).

### Materials availability

This study did not generate new unique reagents.

### Data and code availability

The cryo-EM maps of the nucleosome and the CPDH reported in this paper have been deposited in the Electron Microscopy DataBank (EMDB), under the accession codes EMD-65715 and EMD-65716, respectively. The atomic model of the CPDH has been deposited in the Protein DataBank (PDB), under the accession code PDB: 9W74. This paper does not report original code. Any additional information required to reanalyze the data reported in this paper is available from the lead contact upon request.

## Acknowledgments

We thank J.-H. Min for sharing the pET29a-YS14 plasmid. We would also like to express our deepest gratitude to M. Dacher and Y. Takeda (University of Tokyo) for their assistance. This work was supported in part by 10.13039/501100001691JSPS KAKENHI grant numbers JP23H05475 (to H.K.), JP24H02319 (to H.K.), JP24H02328 (to H.K.), and JP25K18403 (to C.H.H.). This work was supported partly by JST ERATO grant JPMJER1901 (to H.K.), JST CREST grant JPMJCR24T3 (to H.K.), and Research Support Project for Life Science and Drug Discovery (BINDS) grant numbers JP25ama121009 (to H.K.) and JP25ama121002 (to Y.T.).

## Author contributions

Conceptualization, C.H.H., Y.T., and H.K.; data curation, C.H.H., M.O., and Y.T.; formal analysis, C.H.H.; funding acquisition, C.H.H., Y.T., and H.K.; investigation, C.H.H., Y.K., and M.O.; methodology, C.H.H.; project administration, H.K.; supervision, Y.T. and H.K.; validation, C.H.H. and Y.T.; visualization, C.H.H.; writing – original draft, C.H.H.; writing – review and editing, Y.T. and H.K.

## Declaration of interests

The authors declare no competing interests.

## Declaration of generative AI and AI-assisted technologies in the writing process

During the preparation of this work, the authors used ChatGPT, a Large Language Model developed by OpenAI, in a strictly auxiliary role to support the writing process. This included assistance with phrasing and grammar correction. After using ChatGPT, the authors reviewed and edited the content as needed and take full responsibility for the content of the published article.

## STAR★Methods

### Key resources table

**Table:** 

REAGENT or RESOURCE	SOURCE	IDENTIFIER
**Bacterial and virus strains**		

*E. coli* (BL21(DE3))	Novagen (Merck)	Cat#69450

**Chemicals, peptides, and recombinant proteins**		

Proteinase K, recombinant PCR grade	Roche (Merck)	Cat#03115828001
Micrococcal Nuclease (MNase)	TaKaRa	Cat#2910A
PL2-6 single-chain variable fragment (scFv)	Oishi et al.^23^	–

**Deposited data**		

Xenpous nucleosome reconstituted in *E. coli*	This paper	EMDB: EMD-65715
Xenopus close-packed di-hexasome (CPDH)	This paper	EMDB: EMD-65716 PDB: 9W74
Crystal structure of the Xenopus nucleosome	Vasudevan et al.^11^	PDB: 3LZ0
Cryo-EM structure of the human H3-H4 octasome (closed form)	Nozawa et al.^20^	PDB: 7X57

**Recombinant DNA**		

pET29a-YS14 plasmid	Addgene	Cat#66890; RRID: Addgene_66890
pET15b-PL2-6 scFv^23^ (linker peptides: (GGGGS)_4_)	Oishi et al.^23^	–

**Software and algorithms**		

EPU software 3.6.0.6389	Thermo Fisher Scientific	–
CryoSPARC v4.7.1	Punjani et al.^15^	https://cryosparc.com
UCSF ChimeraX-1.8/1.10	Goddard et al.^17^	https://www.cgl.ucsf.edu/chimerax/
COOT 0.9.8.7	Emsley et al.^26^	https://www2.mrc-lmb.cam.ac.uk/personal/pemsley/coot/
Phenix 1.21.2	Afonine et al.^27^	http://www.phenix-online.org
ISOLDE 1.8	Croll^28^	https://isolde.cimr.cam.ac.uk/what-isolde/

**Other**		

VP-050N sonicator	TAITEC	Cat#0079435-000
Ni-NTA agarose beads	QIAGEN	Cat#30250
PD MiniTrap G-25 column	Cytiva	Cat#28918007
HiLoad 26/600 Superdex 75 pg	Cytiva	Cat#28989334
Quantifoil R1.2/1.3 200-mesh Cu	Quantifoil	Cat#M2955C-1
Vitrobot Mark IV	Thermo Fisher Scientific	N/A
Krios G4 transmission electron microscope	Thermo Fisher Scientifc	–
K3 direct electron detector (with a BioQuantum energy filter)	Gatan	–

### Experimental model and study participant details

Xenopus histones H2A, H2B, H3, and H4 were expressed in *E. coli* BL21 (DE3) strain. The single-chain variable fragment (scFv), derived from mouse PL2-6 antibody, was also expressed in *E. coli* BL21 (DE3) strain.

### Method details

#### Plasmid transformation and *E. coli* culture

To co-express all four core histones in *E. coli*, we used the pET29a-YS14 plasmid, which contains a polycistronic construct encoding *Xenopus laevis* histones (S-tag-His₆-H2A, H2B, H3, and H4-His₆). pET29a-YS14 was a gift from Jung-Hyun Min (Addgene plasmid # 66890; http://n2t.net/addgene:66890; RRID:Addgene_66890).^10^ The plasmid was transformed into *E. coli* BL21(DE3) cells, using standard heat-shock transformation. Following recovery in SOC medium with shaking at 1,000 rpm at 37°C for 1 h by an MBR-022 maximizer (TAITEC), the cells were plated on LB agar containing kanamycin (35 μg/mL) and incubated overnight at 37°C.

A single colony was picked and inoculated into 5 mL of LB medium supplemented with kanamycin (35 μg/mL) and grown overnight at 37°C with shaking at 170 rpm (horizontal shake mode) by a BR-13FP BioShaker (TAITEC). The next day, two flasks containing 100 mL LB medium, supplemented with kanamycin (35 μg/mL), were inoculated with the overnight culture (2 mL per flask). Cultures were incubated at 37°C with shaking at 180 rpm by a BR-40UL BioShaker (TAITEC) until the optical density at 600 nm (OD₆₀₀) reached 0.7. At this point, one flask was induced with 400 μM IPTG, while the other was left uninduced. Both cultures were then incubated overnight at 37°C with shaking at 180 rpm by a BR-40UL BioShaker (TAITEC).

The cells were harvested by centrifugation and resuspended in 10 mL of lysis buffer [50 mM Tris-HCl (pH 8.0), 500 mM NaCl, 10 mM 2-mercaptoethanol, 5% glycerol, and 0.1 mM PMSF]. The resuspended cells were then aliquoted into eleven 1 mL tubes for each condition (22 tubes in total). One aliquot from each condition was set aside for histone expression analysis (see next section), while the remaining aliquots were flash-frozen in liquid nitrogen and stored at −80°C until further use for MNase treatment and nucleosome purification.

#### Histone expression check

To evaluate the expression levels of the four histones in *E. coli*, we performed an SDS-PAGE analysis based on previously described work.^7^ Briefly, 1 mL of cultured cells from each condition was lysed using a VP-050N sonicator (TAITEC). From each lysate, 100 μL was subjected to centrifugation, after which 90 μL of the supernatant was transferred into a new tube, and the pellet was resuspended in 80 μL of lysis buffer. Then, 20 μL of each fraction (supernatant and pellet) was mixed with 2× SDS-PAGE loading buffer and heated at 95°C for 10 min. Finally, 4 μL of each sample was loaded onto an 18% SDS-PAGE gel. Protein bands were visualized by Coomassie Brilliant Blue (CBB) staining (Figure 1B).

#### MNase treatment and DNA fragment analysis

MNase treatment was performed based on the previously described ecMNase method (Figure 1A).^7^ Briefly, a frozen 1 mL cell aliquot (which corresponds to 9.09 mL of the originally cultured cells) was thawed, centrifuged, and resuspended in 1 mL of buffer B [25 mM Tris-HCl (pH 8.0) and 15% sucrose]. To generate protoplasts, the sample was centrifuged again and resuspended in 1 mL of buffer A [25 mM Tris-HCl (pH 8.0), 15% sucrose, 1 mM EDTA (pH 8.0), and 0.21 mg/mL lysozyme], and then incubated at room temperature for 17 min. Afterward, the sample was centrifuged, and the pellet was resuspended in 1 mL of buffer B. This centrifugation and resuspension step was repeated twice (three washes in total). After the final centrifugation, the pellet was resuspended in 200 μL of MNase buffer [10 mM Tris-HCl (pH 8.0), 25% sucrose, 50 mM NaCl, 5 mM MgCl_2_, 1 mM CaCl_2_, 1 mM 2-mercaptoethanol, 0.1% NP-40]. MNase (TaKaRa Bio; 20 U/μL) was added to a final concentration of 800 U/mL (8 μL added), and the mixture was incubated at 37°C for 15 min. The reaction was quenched by adding 10 μL of 0.5 M EDTA (pH 8.0), followed by centrifugation. The supernatant was collected for subsequent DNA fragment analysis and Ni-NTA purification.

For the DNA fragment analysis, 5 μL of the supernatant was mixed with 1 μL of Proteinase K solution [9.3 mg/mL Proteinase K, recombinant PCR grade (Roche) and 1.67% SDS], and incubated at 37°C for 15 min. The sample was then analyzed by 6% native PAGE in 0.5× TBE (Figure 1C).

#### Nucleosome purification and quality check

In a 1.5 mL microcentrifuge tube, the supernatant from the previous section was mixed with 50 μL of Ni-NTA agarose beads (QIAGEN) that had been pre-washed with 400 μL of MNase buffer. The mixture was rotated at 4°C for 1 h to allow the binding of S-tag-His₆-H2A and H4-His₆ to the beads. The beads were then centrifuged, and the unbound fraction (flowthrough) was removed. The beads were washed once with 250 μL of Ni-NTA wash buffer [50 mM Tris-HCl (pH 8.0), 100 mM NaCl, 1 mM DTT, 5% glycerol, and 10 mM imidazole], and the bound proteins were eluted with 250 μL of Ni-NTA elution buffer [50 mM Tris-HCl (pH 8.0), 100 mM NaCl, 1 mM DTT, 5% glycerol, and 300 mM imidazole].

Subsequently, buffer exchange was performed using a PD MiniTrap G-25 column (Cytiva). The sample was eluted in TCS buffer [20 mM Tris-HCl (pH 7.5) and 1 mM DTT], concentrated to approximately 30 μL, and stored at 4°C. The final nucleosome quality was assessed by 18% SDS-PAGE and 6% native PAGE in 0.5× TBE (Figures 1D and 1E). The DNA concentration of the purified nucleosome was around 0.2 mg/mL, corresponding to approximately 2.1 μM assuming a DNA length of 145 bp. Therefore, from a starting culture of 9.09 mL *E. coli* cells, this yields roughly 62 pmol of nucleosomes—sufficient to perform one PL2-6 binding assay and prepare about eight cryo-EM grids, as described in the following two sections.

#### PL2-6 binding assay

PL2-6 (scFv) was used in this study and prepared as previously described.^23^ Briefly, the scFv was expressed in the *E. coli* strain BL21 (DE3) by induction with 500 μM IPTG. The cells were harvested, sonicated, and centrifuged, and the pellet was washed several times with wash buffer [50 mM Tris–HCl (pH 8.0), 100 mM NaCl, 1% Triton X-100, and 1 M urea]. Subsequently, the pellet was washed with phosphate-buffered saline (PBS), and denaturing buffer [100 mM Tris and 6 M urea] was then added to the pellet. This mixture was stirred overnight at 4°C; the next day, following centrifugation, the supernatant was mixed with Ni-NTA agarose resin. The beads were then washed with denaturing buffer supplemented with 20 mM imidazole and eluted with denaturing buffer supplemented with 400 mM imidazole. The collected sample was then dialyzed against denaturing buffer, which was gradually replaced by refolding buffer [50 mM Tris–HCl (pH 7.5), 150 mM NaCl, and 1 mM EDTA]. The sample was further dialyzed against fresh refolding buffer, and then purified on a HiLoad 26/600 Superdex 75 pg (Cytiva) column.

The PL2-6 binding assay was performed by mixing 0.20 μM nucleosome (estimated from DNA concentration, assuming a nucleosome DNA length of 145 bp) with 0.20, 0.40, 0.60, or 0.80 μM PL2-6, in a solution containing 26 mM Tris-HCl (pH 7.5), 30 mM NaCl, 0.2 mM EDTA, and 0.8 mM DTT. The mixture was incubated on ice for 15 min and then analyzed by 6% native PAGE in 0.5× TBE (Figure 2A).

#### Cryo-EM grid preparation and data collection

Prior to cryo-EM grid preparation, 1.93 μM nucleosome (estimated from DNA concentration, assuming a nucleosome DNA length of 145 bp) was mixed with 5.77 μM PL2-6, in a solution containing 22.5 mM Tris-HCl (pH 7.5), 12.3 mM NaCl, 0.92 mM EDTA, and 0.92 mM DTT. Based on our previous experience, high NaCl concentrations can lead to nucleosome stacking in cryo-EM micrographs; therefore, the NaCl concentration was minimized. The mixture was kept on ice until plunge-freezing.

Grids were prepared using a Vitrobot Mark IV (Thermo Fisher Scientific), operating at 4°C and 100% humidity. A 2.5 μL aliquot of the sample was applied to a glow-discharged Quantifoil R1.2/1.3 200 mesh Cu grid. The grids were blotted under the following conditions: blot time, 5 s; wait time, 0 s; blot force, 0; blot total, 1; and drain time, 0 s. Grids were then plunge-frozen into liquid ethane for vitrification. Grids were stored in liquid nitrogen until screening and data collection.

The grids were loaded into a Krios G4 transmission electron microscope (Thermo Fisher Scientific) for screening and data collection. Cryo-EM images were acquired using a K3 direct electron detector equipped with a BioQuantum energy filter (Gatan) with a 20 eV slit width, and operated through the EPU software (Thermo Fisher Scientific) with the following settings: pixel size, 1.06 Å; defocus range, −1.25 to −2.25 μm; and total dose, 60.1 e⁻/Å² over 40 frames. A total of 7,103 movies were collected. More details are provided in Table S1.

#### Cryo-EM image processing

The movies were imported into CryoSPARC (v4.7.1)^15^ for single particle analysis (Figure S1). Motion correction was performed with Patch Motion Correction, followed by CTF estimation using Patch CTF Estimation. Low-quality micrographs were discarded using the Curate Exposures job. Particles were picked using Blob Picker with the following parameters: minimum particle diameter, 100 Å; maximum particle diameter, 200 Å; and minimum separation distance, 0.5 diameters. Picked particles were visually inspected and manually thresholded using the Inspect Particle Picks job. Particles were extracted with a 200-pixel box size (212 Å), Fourier cropped to 100 pixels, and subjected to one round of 2D classification to remove junk particles.

An Ab-initio Reconstruction job was used to generate four initial volumes. Notably, two of the four volumes resembled nucleosomes and hexasomes. All four volumes (two copies each) were then used as input references for Heterogeneous Refinement, which successfully identified one nucleosome-like class and one hexasome-like class for downstream processing.

Particles from these two classes were re-extracted at full resolution (without Fourier cropping), followed by Homogeneous Refinement and Local CTF Refinement, yielding maps at 2.83 Å (nucleosome) and 2.93 Å (hexasome-like). These maps were then subjected to Reference-Based Motion Correction, and the corrected particles were refined using Non-uniform Refinement, resulting in improved maps at 2.66 Å (nucleosome) and 2.69 Å (hexasome-like). Further rounds of Local CTF Refinement and Non-uniform Refinement yielded final maps of 2.62 Å and 2.70 Å, respectively.

In the hexasome-like map, weak densities of the H2A-H2B dimer could still somehow be observed. To rule out the possibility that nucleosome particles are also present in this particle stack, a few more rounds of 3D classification were performed. Several nucleosome-like classes were obtained and merged with the original 2.62 Å nucleosome particle stack. This final nucleosome particle stack was refined using Non-uniform Refinement, yielding a final nucleosome map at 2.56 Å resolution.

To verify that the hexasome-like particles were truly hexasomes and not subunits of a larger, non-canonical nucleosome structure, the remaining particles from the hexasome-like group were re-extracted using a larger box size (320 pixels; 339.2 Å). An Ab-initio Reconstruction job on this particle stack revealed two classes: one corresponding to a hexasome-like structure and another with a hexasome and additional density. Both volumes were used as input references for a Heterogeneous Refinement job, which identified a distinct class corresponding to the close-packed di-hexasome (CPDH).

Because some residual, ambiguous density was still observed in the hexasome-like classes on the H2A-H2B-deficient side, we cannot conclusively determine whether these represent true hexasomes. At this stage, the number of hexasome-like particles was low. Given that hexasome structures have been previously reported, we chose to focus on the CPDH class, which has not been reported. Particles from the CPDH class were re-centered using Volume Alignment Tools and re-extracted with the same 320-pixel box size. These particles were refined, locally motion-corrected, and subjected to 3D classification. One class displaying clear density for both hexasomes was selected and refined using Non-uniform Refinement, yielding the final CPDH map.

More details can be found in Figures S1 and S2 and Table S1.

#### Model building and refinement

The structural model of the CPDH was constructed primarily by using two copies of the *Xenopus laevis* nucleosome crystal structure (PDB: 3LZ0). These models were individually fitted into the CPDH cryo-EM density map, using ChimeraX.^17^ The additional H2A-H2B dimers and associated DNA segments were removed from both nucleosome models to reflect the hexasome composition. To connect the central DNA between the two hexasomes, a short DNA fragment was extracted from the overlapping tri-nucleosome structure (PDB: 8IHL) and manually trimmed to fit between the two hexasomes. The combined model was refined by real-space refinement in Phenix^27^ and further adjusted manually using COOT^26^^,^^29^ and ISOLDE.^28^ Because the DNA contains various sequences from the *E. coli* genome, a poly-dA/dT sequence was used for model building. More details can be found in Table S1.
