# Supplementary material for: A method for cryo-EM analysis of eukaryotic nucleosomes reconstituted in bacterial cells
Source: iScience. 2025 Dec 16;29(1):114453. doi: 10.1016/j.isci.2025.114453 (PMC12803842; doi:10.1016/j.isci.2025.114453)
Supplement: Document S1. Figures S1–S3 and Table S1 [file mmc1.pdf]

iScience, Volume *29*

Supplemental information

A method for cryo-EM analysis of eukaryotic nucleosomes reconstituted in bacterial cells

Cheng-Han Ho, Yuki Kobayashi, Mitsuo Ogasawara, Yoshimasa Takizawa, and Hitoshi Kurumizaka

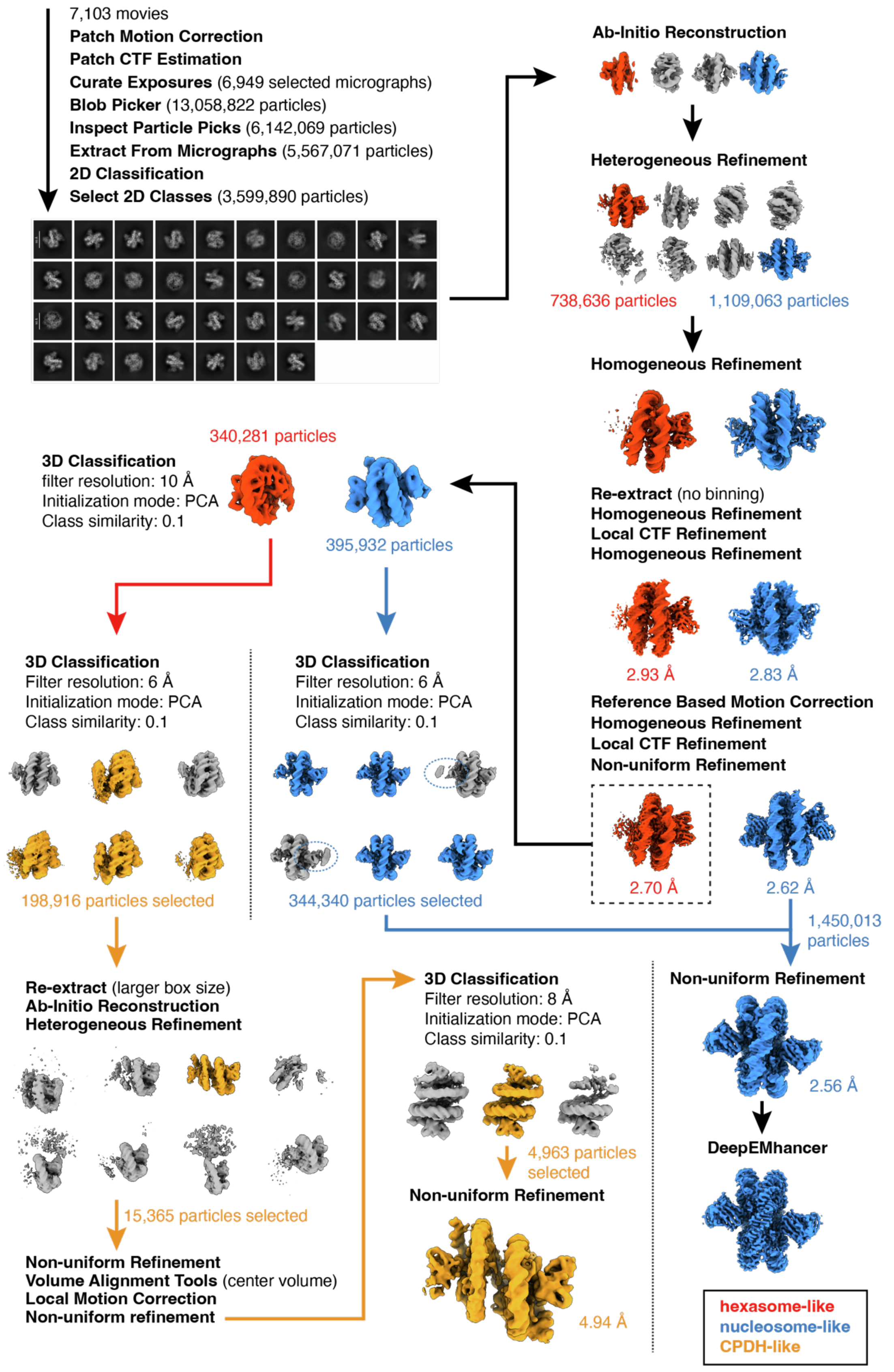
7,103 movies
Patch Motion Correction
Patch CTF Estimation
Curate Exposures (6,949 selected micrographs)
Blob Picker (13,058,822 particles)
Inspect Particle Picks (6,142,069 particles)
Extract From Micrographs (5,567,071 particles)
2D Classification
Select 2D Classes (3,599,890 particles)
Ab-Initio Reconstruction
Heterogeneous Refinement
738,636 particles
1,109,063 particles
Homogeneous Refinement
Re-extract (no binning)
Homogeneous Refinement
Local CTF Refinement
Homogeneous Refinement
2.93 Å
2.83 Å
Reference Based Motion Correction
Homogeneous Refinement
Local CTF Refinement
Non-uniform Refinement
2.70 Å
2.62 Å
340,281 particles
3D Classification
filter resolution: 10 Å
Initialization mode: PCA
Class similarity: 0.1
395,932 particles
3D Classification
Filter resolution: 6 Å
Initialization mode: PCA
Class similarity: 0.1
198,916 particles selected
3D Classification
Filter resolution: 6 Å
Initialization mode: PCA
Class similarity: 0.1
344,340 particles selected
1,450,013
particles
Re-extract (larger box size)
Ab-Initio Reconstruction
Heterogeneous Refinement
15,365 particles selected
Non-uniform Refinement
Volume Alignment Tools (center volume)
Local Motion Correction
Non-uniform refinement
3D Classification
Filter resolution: 8 Å
Initialization mode: PCA
Class similarity: 0.1
4,963 particles
selected
Non-uniform Refinement
4.94 Å
Non-uniform Refinement
2.56 Å
DeepEMhancer
hexasome-like
nucleosome-like
CPDH-like

Figure S1. Cryo-EM single particle analysis workflow by CryoSPARC.

The hexasome-like, nucleosome-like, and CPDH-like particles are colored red, blue, and orange, respectively. In the center panel, in which the 395,932 nucleosome-like particles were subjected to 3D classification, particles were selected based on the clarity of the PL2-6 density. The circled regions indicate the unclear PL2-6 densities.

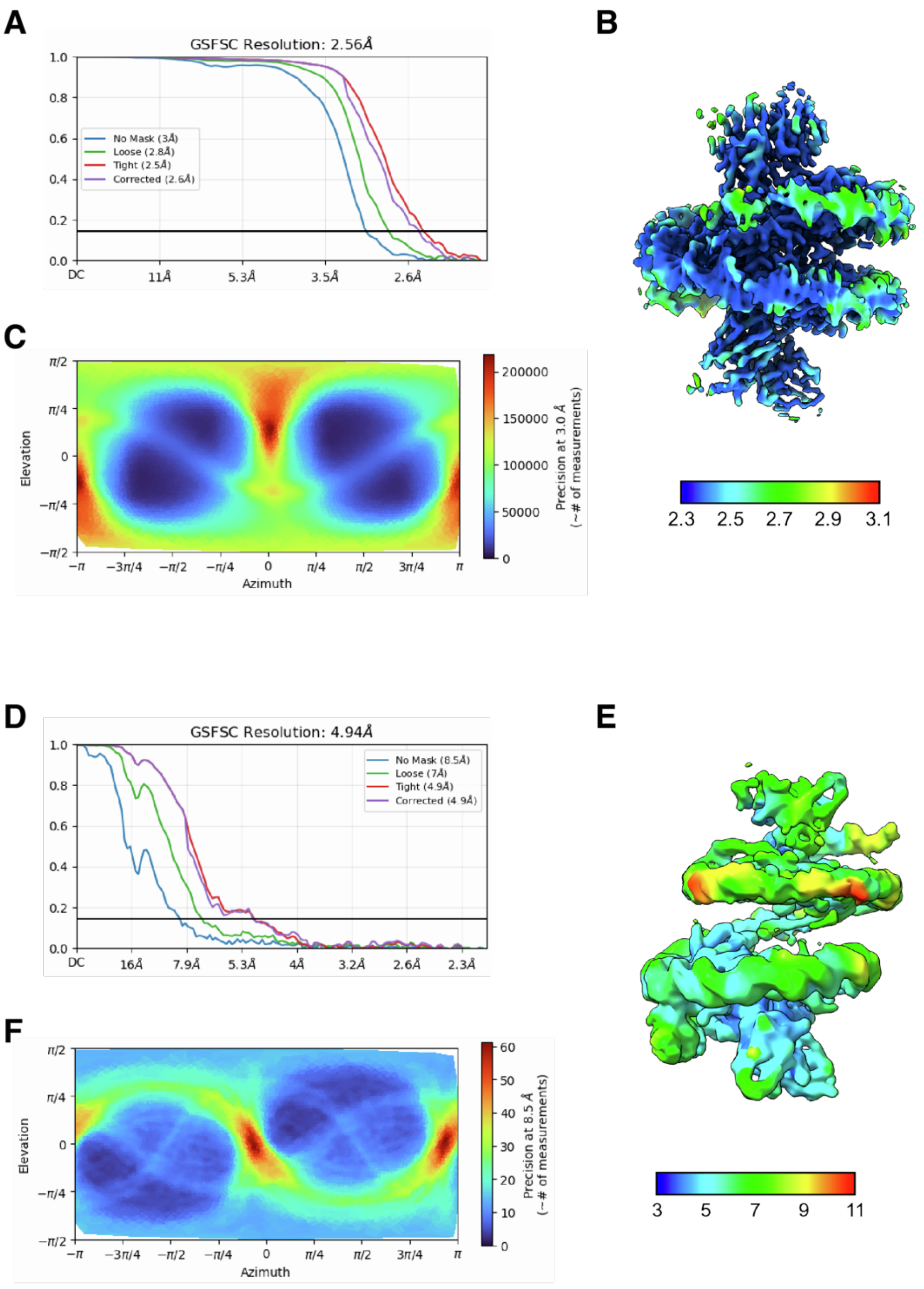

Figure S2. Details of the cryo-EM maps of the PL2-6-nucleosome complex and the PL2-6-close-packed di-hexasome (CPDH) complex.

(A-C) Fourier shell correlation curves (A), local resolutions (B), and Euler angle distributions (C) of the PL2-6-nucleosome complex. (D-F) Fourier shell correlation curves (D), local resolutions (E), and Euler angle distributions (F) of the PL2-6-CPDH complex.

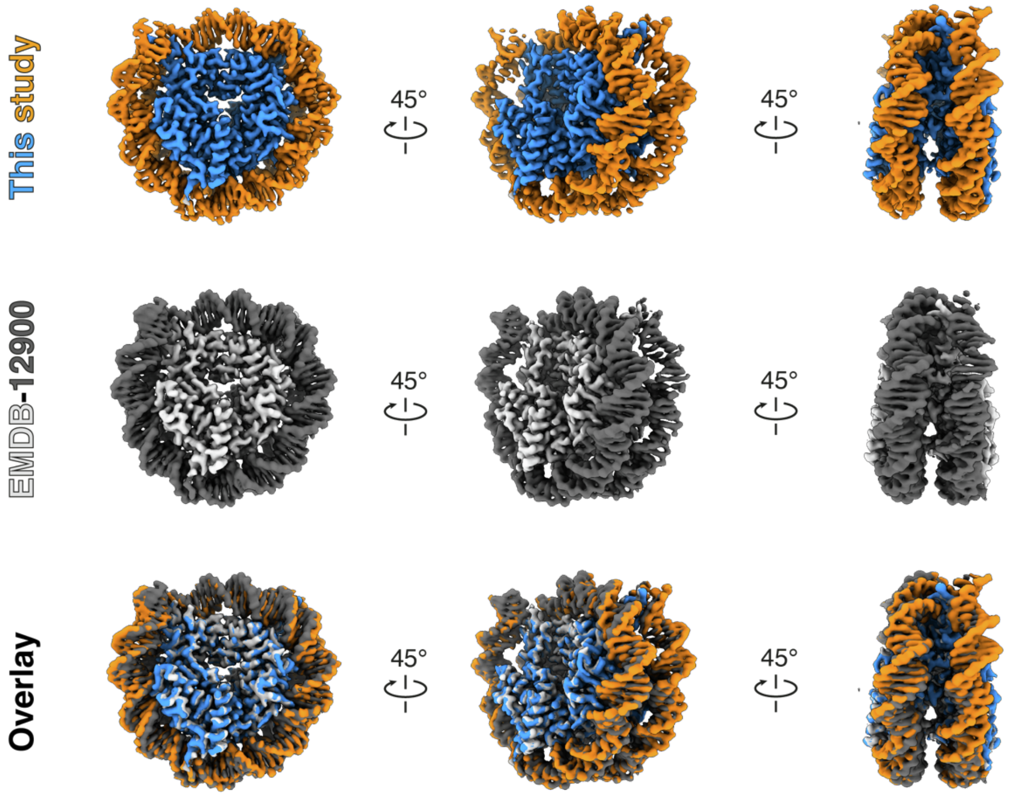

Figure S3. Comparison between the cryo-EM map of the E. coli–derived nucleosome (this study) and that of an *in vitro* reconstituted nucleosome (EMDB-12900).

Table S1. Details of cryo-EM data collection, image processing, and model building.

Sample	Close-packed di-hexasome (CPDH)	Nucleosome
	EMD-65716	EMD-65715
	PDB: 9W74	[redacted]
**Data collection**		
Electron microscope	Krios G4	
Camera	K3 BioQuantum	
Pixel size (Å/pixel)	1.06	
Decocus range (µm)	-1.25 to -2.25	
Total dose (e-/Å^2)	60.1	
Movie frames (no.)	40	
Total micrographs (no.)	7,103	
**Reconstruction**		
Software	CryoSPARC	
Particles in the final map (no.)	4,963	1,450,013
Symmetry	C1	C1
Final resolution (Å^2)	4.94	2.56
FSC threshold	0.143	0.143
Map sharpening B factor (Å^2)	-53.7	N/A (DeepEMhancer)
**Model building**		[redacted]
Software	Coot	[redacted]
**Refinement**		[redacted]
Software	Phenix	[redacted]
**Model composition**		[redacted]
Protein	1,099	[redacted]
Nucleotide	414	[redacted]
**Validation**		[redacted]
MolProbity score	2.20	[redacted]
Clash score	23.47	[redacted]
**R.m.s. deviations**		[redacted]
Bond lengths (Å)	0.006	[redacted]
Bond angles (˚)	0.769	[redacted]
**Ramachandran plot**		[redacted]
Favored (%)	95.07	[redacted]
Allowed (%)	4.93	[redacted]
Outliers (%)	0.00	[redacted]
